# TLR Ligands Induce Antiviral Responses in Chicken Macrophages

**DOI:** 10.1371/journal.pone.0105713

**Published:** 2014-08-28

**Authors:** Neda Barjesteh, Shahriar Behboudi, Jennifer T. Brisbin, Alexander Ian Villanueva, Éva Nagy, Shayan Sharif

**Affiliations:** 1 Department of Pathobiology, University of Guelph, Guelph, Ontario, Canada; 2 The Pirbright Institute, Compton Laboratory, Newbury, United Kingdom; University of Saskatchewan, Canada

## Abstract

Chicken macrophages express several receptors for recognition of pathogens, including Toll-like receptors (TLRs). TLRs bind to pathogen-associated molecular patterns (PAMPs) derived from bacterial or viral pathogens leading to the activation of macrophages. Macrophages play a critical role in immunity against viruses, including influenza viruses. The present study was designed to test the hypothesis that treatment of chicken macrophages with TLR ligands reduces avian influenza replication. Furthermore, we sought to study the expression of some of the key mediators involved in the TLR-mediated antiviral responses of macrophages. Chicken macrophages were treated with the TLR2, 3, 4, 7 and 21 ligands, Pam3CSK4, poly(I:C), LPS, R848 and CpG ODN, respectively, at different doses and time points pre- and post-H4N6 avian influenza virus (AIV) infection. The results revealed that pre-treatment of macrophages with Pam3CSK4, LPS and CpG ODN reduced the replication of AIV in chicken macrophages. In addition, the relative expression of genes involved in inflammatory and antiviral responses were quantified at 3, 8 and 18 hours post-treatment with the TLR2, 4 and 21 ligands. Pam3CSK4, LPS and CpG ODN increased the expression of interleukin (IL)-1β, interferon (IFN)-γ, IFN-β and interferon regulatory factor (IFR) 7. The expression of these genes correlated with the reduction of viral replication in macrophages. These results shed light on the process of immunity to AIV in chickens.

## Introduction

Macrophages are important cells of the innate immune system that play a critical role in the initiation of immune responses against pathogens, such as influenza viruses [1]–[4]. Functions of macrophages include phagocytosis, cytokine and chemokine production, secretion of antimicrobial factors and peptides and antigen presentation [5]–[7]. Macrophages express several receptors for recognition of pathogens, including Toll-like receptors (TLRs). TLRs bind to pathogen-associated molecular patterns (PAMPs) or synthetic ligands leading to activation of macrophages.

Influenza viruses can infect macrophages and dendritic cells [8], leading to the production of cytokines and chemokines in response to the infection [9]. In chickens, macrophages produce pro-inflammatory cytokines such as interleukin (IL)-1β, IL-6 and IL-8 in response to avian influenza virus infection [10], subsequently attracting other cells of the immune system, such as heterophils, to the site of infection [11]. In addition to pro-inflammatory cytokines, chicken macrophages produce type I interferons (IFNs) upon infection [12]. From studies in mammals, it is known that after binding of IFNs to their receptors, the Janus kinase and signal transducer and activator of transcription (JAK-STAT) signalling pathway is activated, which in turn up-regulates the expression of interferon-stimulated genes (ISGs). ISGs encode a wide variety of proteins, such as RNA-activated protein kinase (PKR), oligoadenylate synthase (OAS) and ribonuclease L (RNase L), that play crucial roles in immune responses against viral infections [8], [13], [14]. IFNs and ISGs can inhibit the replication of the virus by preventing entry of virus into host cells, abolishing translation processes, attaching to viral RNA, sequestering viral proteins and regulating host antiviral responses. In addition, ISGs modulate adaptive immune responses by affecting the secondary CD8+ T cell responses or facilitating T cell receptor (TCR)-mediated GATA3 activation to produce T helper (Th)2 cytokines [8], [15]–[17].

At least 10 TLR genes have been identified in chickens, including TLR1A, 1B, 2A, 2B, 3, 4, 5, 7, 15 and 21 [18]. The immunomodulatory role of TLR ligands has been demonstrated in chickens. Treatment of chicken cells, such as splenocytes, macrophages and monocytes with TLR2, 3, 4 and 21 ligands significantly up-regulates the expression of pro-inflammatory cytokines, such as IL-1β, IL-6 and IL-8 [19]–[21]. In addition to pro-inflammatory responses, TLR ligand administration may lead to induction of biased Th responses. It has recently been demonstrated that ligands for TLR2, 4 induce Th1-like and Th2-like responses while ligands for TLR21 induce Th1-like responses in chickens [19], [21], [22]. TLR ligands are also known to induce antiviral responses, including the induction of type I IFNs in chickens [22], [23]. Considering the immunostimulatory activities of TLR ligands, these molecules have been successfully used for conferring immunity on the host against viral and bacterial pathogens [24]–[27]. In mammals, it is known that TLR ligands can exert antiviral activity against influenza viruses and cause protection against influenza virus infection [25], [28]. Previous studies demonstrated that prophylactic treatment of chickens with polyI:C, CpG ODN and LPS can reduce shedding of low pathogenic AIV [22]. However, little information is available about the antiviral mechanisms of these ligands in chickens and their underlying mechanisms of action against AIV. Therefore, the objective of the present study was to examine the potential of TLR ligands to limit low pathogenic AIV replication in avian macrophages. We hypothesized that TLR ligands induce antiviral activities in chicken macrophages. These antiviral activities may be responsible for restricting the replication of AIV in these cells.

## Materials and Methods

### Avian influenza virus

The A/Duck/Czech/56 (H4N6), a low pathogenic avian influenza virus (LPAIV), was used in the study. The virus was propagated in 11-day-old embryonated chicken eggs by inoculation through the allantoic cavity [29]. Briefly, embryonated chicken eggs were candled and embryos were inoculated with 100 µl of allantoic fluid containing 0.2 hemagglutination units (HAU) of H4N6. The allantoic fluid was harvested after 72 h and the virus titre was determined using end-point dilution in Madin-Darby canine kidney (MDCK) cells and expressed as 50% tissue culture infective dose (TCID_50_)/ml according to the Reed-Muench formula (WHO Manual on Animal Influenza Diagnosis and Surveillance, 2002) [30].

### Cell culture

The chicken macrophage cell line (MQ-NCSU) was kindly provided by Dr. Juan Carlos Rodriguez (University of Prince Edward Island, Canada). This cell line was derived from spleen cells of a chicken infected with the JM/102W strain of Marek's disease virus [31]. The cells were maintained in 1:1 combination of Mc Coy's 5A modified medium and L-15 Leibovitz medium supplemented with 8% fetal bovine serum (FBS), 10% chicken serum, 1% tryptose phosphate broth, 1% sodium pyruvate, 2 mM L-glutamine, 200 U/ml penicillin, 80 µg/ml streptomycin, and 50 µg/ml gentamicin at 41°C and 5% CO_2_ in a humidified incubator.

### Avian influenza virus infection of cells

MQ-NCSU cells were seeded into 24-well cell culture plates at a viable cell density (determined by Trypan blue exclusion) of 5 × 10^5^ cells/ml in DMEM containing 10% FBS, 200 U/ml penicillin, and 80 µg/ml streptomycin for 18 hours. Cells were washed with DMEM and medium was replaced with DMEM supplemented with 200 U/ml penicillin, 80 µg/ml streptomycin, 50 µg/ml gentamicin, 25 mM HEPES, 7.5% BSA and 1 µg/µl trypsin-TPCK. Cells were infected with a multiplicity of infection (MOI) of 1 with H4N6 AIV. In a preliminary study, different MOI were tested (0.5, 1, 5 and 10) and it was determined that a MOI of 1 was ideal for H4N6 AIV replication in chicken macrophages (data not shown). Subsequent to infection, cells were washed two times after 2 hours and fresh medium was added to the culture. The virus titer in supernatants was measured using a TCID_50_ assay at different time points (0, 6, 16, 24, 32 and 42 hours) post-infection.

### TLR ligands

Pam3CSK4 (synthetic triacylated lipoprotein), polyI:C and R848 were purchased from Invivogen (San Diego, California, USA). Synthetic class A CpG ODN 2216 [5′- GGGGGACGA:TCGTCGGGGGG-3′], synthetic class B CpG ODN 2007 [5′- TCGTCGTTGTCGTTTTGTCGTT- 3′], synthetic class B CpG ODN 1826 [ 5′- TCCATGACGTTCCTGACGTT- 3′], synthetic class C CpG ODN 2395 [5′- TCGTCGTTT-TCG-GCGCGCGCCG- 3′], non-CpG ODN [5′ -TGCTGCTTGTGCTTTTGTGCTT- 3′], lipopolysaccharides (LPS) from *Escherichia coli* 0111:B4 and LPS from *E. coli* 026:B6 were purchased from Sigma–Aldrich (Oakville, Ontario, Canada). These ligands were selected as they have previously been shown to stimulate chicken TLRs [18], [19].

### Macrophage treatment with TLR ligands and cell infection with avian influenza virus

MQ-NCSU cells were seeded into 24-well cell culture plates at a viable cell density (determined by Trypan blue exclusion) of 5 × 10^5^ cells/ml in DMEM containing 10% FBS, 200 U/ml penicillin and 80 µg/ml streptomycin for 18 hours. Subsequently, cells were stimulated with three different doses of TLR ligands (Table 1) and their ability to stimulate nitric oxide (NO) production in culture supernatants which were collected 48 hours post-stimulation, as measured by Griess assay (Promega, Madison, WI) according to the manufacturer's instructions. The amount of nitrite in the each experimental sample was quantified by comparing to the standards available in the Griess assay kit. Various doses of TLR ligands were selected based on the results of previous studies in chickens and manufacturer's recommendations [4], . The doses are within the range that has been shown to activate macrophages or monocytes [36]–[38]. We analysed NO production to screen the ability of different doses of TLR ligands to induce macrophage activation. Analysis of NO production is known to be a reliable method for measuring macrophage activation [39], [40].

**Table 1:** TLR ligands and their corresponding doses in MQ-NCSU cells.

TLR ligand	TLR	High dose (µg/µl)	Intermediate dose (µg/µl)	Low dose (µg/µl)
Pam3CSK4	TLR2/1	10*	1	0.1
PolyI∶C	TLR3	50*	10	1
LPS (*E. coli* 0111∶B4)	TLR4	10	1*	0.1
LPS (*E. coli* 026∶B6)	TLR4	10	1*	0.1
R848	TLR7	10*	1	0.1
CpG ODN 2216 (class A)	TLR21	10*	1	0.1
CpG ODN 2007 (class B)	TLR21	10*	1	0.1
CpG ODN 1826 (class B)	TLR21	10*	1	0.1
CpG ODN 2395 (class C)	TLR21	10*	1	0.1
Non-CpG ODN		10		

The optimal dose of TLR ligands was determined based on NO production in chicken macrophages.

^*^Determined to be the optimal dose.

To determine the effect of *in vitro* administration of TLR ligands on the replication of H4N6 AIV in chicken macrophages, these cells were treated with the optimum dose (Table 1) of various TLR ligands (determined above) at different time points, including at the time of infection, prior to or after infection with low pathogenic H4N6 AIV as described above. Specifically, the time points included co-administration of TLR ligands and H4N6 AIV as well as treatment 1, 6 and 12 hours prior to infection and 1, 6 and 12 hours post-infection. There were four replicates in each group. Virus titers in the supernatant were determined 14 hours post-infection via the TCID_50_ assay or hemagglutination test (WHO Manual on Animal Influenza Diagnosis and Surveillance) [30].

### Gene expression in chicken macrophages stimulated with TLR ligands

MQ-NCSU cells were seeded into 24-well cell culture plates at a viable cell density (determined by trypan blue exclusion) of 2 × 10^6^ cells/ml in DMEM containing 10% FBS, 200 U/ml penicillin and 80 µg/ml streptomycin for 18 hours. Cells were washed with DMEM and the cell culture medium was replaced with DMEM supplemented with 200 U/ml penicillin, 80 µg/ml streptomycin, 50 µg/ml gentamicin, 25 mM HEPES, 7.5% BSA and 1 µg/µl trypsin-TPCK. Cells were stimulated with either class B CpG ODN 1826 (10 µg/ml), Pam3CSK4 (10 µg/ml) or LPS from *E. coli* 026:B6 (1 µg/ml). The control groups were treated with non-CpG ODN (10 µg/mL) or received cell culture medium only. Cells were collected for RNA extraction at 3, 8 and 18 hours post-treatment. There were 6 biological replicates in each group.

### RNA extraction and cDNA synthesis

Total RNA was extracted with Trizol reagent (Life Technologies, Burlington, Ca), according to the manufacturer's recommendations. Total RNA was treated with the DNA Free DNAse kit (Ambion, Austin, TX), and 1 µg of RNA was used for cDNA synthesis using Superscript II First Strand Synthesis kit (Life Technologies, Burlington, Ca) and oligo-dT primers, according to the manufacturer's protocol.

### Real-time PCR

Quantitative real-time PCR was performed on diluted cDNA (1∶10 in DEPC treated water) using SYBR green dye in a LightCycler 480 II (Roche Diagnostics GmbH, Mannheim, DE) as previously described [21], [41]. Briefly, the amplification conditions consisted of pre-incubation for 10 min at 94°C, followed by 45 cycles of 95°C for 10 s, 55–64°C annealing as described in table 2 for each of the primers for 5 s and elongation and signal acquisition (single mode) at 72°C for 10 s. Melting curve analysis was done in three steps; 95°C for 10 s, cooling to 65°C for 1 min and heating to 97°C. Specific sequences of primers were described previously, and are listed in Table 2 [21], [42]–[44].

**Table 2:** Real-time RT-PCR primer sequences for chicken target genes.

Gene Name	Primer sequence	Annealing Temp.(°C)	Reference
β-actin	F: 5'-CAACACAGTGCTGTCTGGTGGTA-3'	60	41
	R: 5'-ATCGTACTCCTGCTTGCTGATCC-3'		
Interferon-α	F: 5'-ATCCTGCTGCTCACGCTCCTTCT-3	64	41
	R: 5'-GGTGTTGCTGGTGTCCAGGATG-3'		
Interleukin-1β	F: 5'-GTGAGGCTCAACATTGCGCTGTA-3	64	41
	R: 5'-TGTCCAGGCGGTAGAAGATGAAG-3'		
Interferon-γ	F: 5'-ACACTGACAAGTCAAAGCCGCACA-3	60	42
	R: 5'-AGTCGTTCATCGGGAGCTTGGC-3'		
Interferon-β	F: 5'-GCCTCCAGCTCCTTCAGAATAC G- 3'	64	43
	R: 5'-CTGGATCTGGTTGAGGAGGCTGT-3'		
2′-5′ Oligoadenylate synthase	F:5'-AGAACTGCAGAAGAACTTTGTC-3'	60	43
	R:5'-GCTTCAACATCTCCTTGTACC-3'		
Interferon regulatory factor 1	F: 5′-ATGAGGATGGAGGAGTCAGCAGA-3′	60	This paper
	R: 5′- CTGGTAGATGTCGTTGGTGCTGT -3′		
Interferon regulatory factor 2	F: 5′- CAGCAGTGAGGAGCAGGTGATAG -3′	60	This paper
	R: 5′- TCTTCATCGCTTGGAACGCTGTC -3′		
Interferon regulatory factor 7	F: 5′- CTCCCCTCCTCCAAAAGCTG -3′	60	This paper
	R: 5′- CTGGGAGCGAAGGAGGAATG -3′		

### Flow cytometry

MQ-NCSU cells (1 × 10^6^ cells/ml) were stimulated with class B CpG ODN 1826 (10 µg/ml), Pam3CSK4 (10 µg/ml) or LPS from *E. coli* 026∶B6 (1 µg/ml), non-CpG ODN (10 µg/ml) or medium only (no stimulation) for 24 hours as described above. The cells were harvested and stained with FITC conjugated anti-chicken major histocompatibility complex (MHC) class II molecules (Clone 2G11-IgG1, Bioscience, Cambridge, UK), Alexa Fluor 647-conjugated mouse anti-chicken CD80 (clone AV82- IgG2a), Alexa Fluor 647-conjugated mouse anti-chicken CD86 (clone AV88- IgG1) or isotype controls (Bioscience, Cambridge, UK). AV82 and AV88 were kind gifts from Dr John R. Young. The cells were washed in PBS and analysed by flow cytometry and FlowJo v10 was used for analysis of the data.

### Statistical analysis

To examine whether TLR ligand treatment of macrophages altered NO production or viral replication, statistical analysis was performed by one-way ANOVA followed by Tukey's post hoc test for multiple comparisons. Statistical analysis of the TCID_50_ data was performed using the two tailed student's t test to compare viral titre between treated groups and the untreated group. Relative expression of all genes was calculated relative to the housekeeping gene β-actin using the LightCycler 480 software (Roche Diagnostics). Relative expression data represent mean fold-change of 6 replicates compared to the medium control group ± standard error. For gene expression, statistical significance was calculated using a two tailed t-test. For all analyses, *P* ≤ 0.05 was considered statistically significant.

## Results

### Low pathogenic avian influenza virus replicates in chicken macrophages

**Figure 1:**
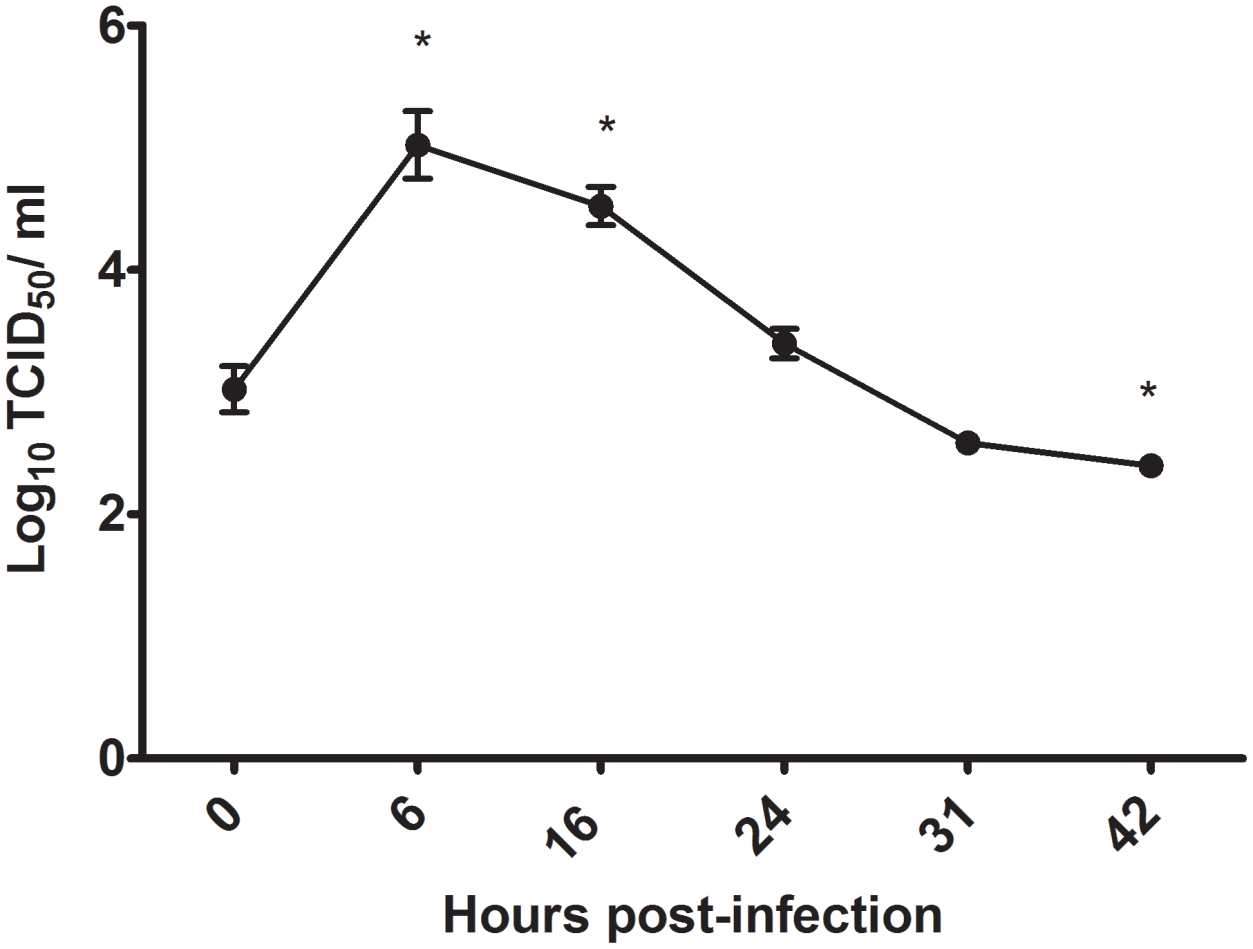
The replication of low pathogenic H4N6 AIV in chicken macrophages (MQ-NCSU cell line). Chicken macrophages were infected with low pathogenic H4N6 AIV at a MOI of 1. Cell supernatants were collected at 0, 6, 16, 24, 31 and 42 hours post-infection. This figure is representative of three separate experiments with four biological replicates per time point. Virus titre is represented by the log_10_ TCID_50_. Significant differences (*P* ≤ 0.05) between the viral titer at a specific time point and the time of infection (time 0) are indicated by an *.

Viral infection and replication were analyzed by assaying infectious virus in cell culture supernatant using the TCID_50_ assay. The results demonstrated that the virus titre was significantly increased by 100 fold at 6 hours post-infection with a log_10_ TCID_50_/mL of 5.02 compared to the time of infection (time 0) with a log_10_ TCID_50_/mL of 3.02. In addition, the virus titre was significantly increased at 16 hours post-infection by 32 fold with a log_10_ TCID_50_/mL of 4.52 compared to the time of infection (Fig. 1). Furthermore, the virus titre was significantly decreased by 5 fold at 42 hours post-infection relative to time of infection (time 0) (Fig. 1).

### TLR ligands induce NO production in chicken macrophages

**Figure 2:**
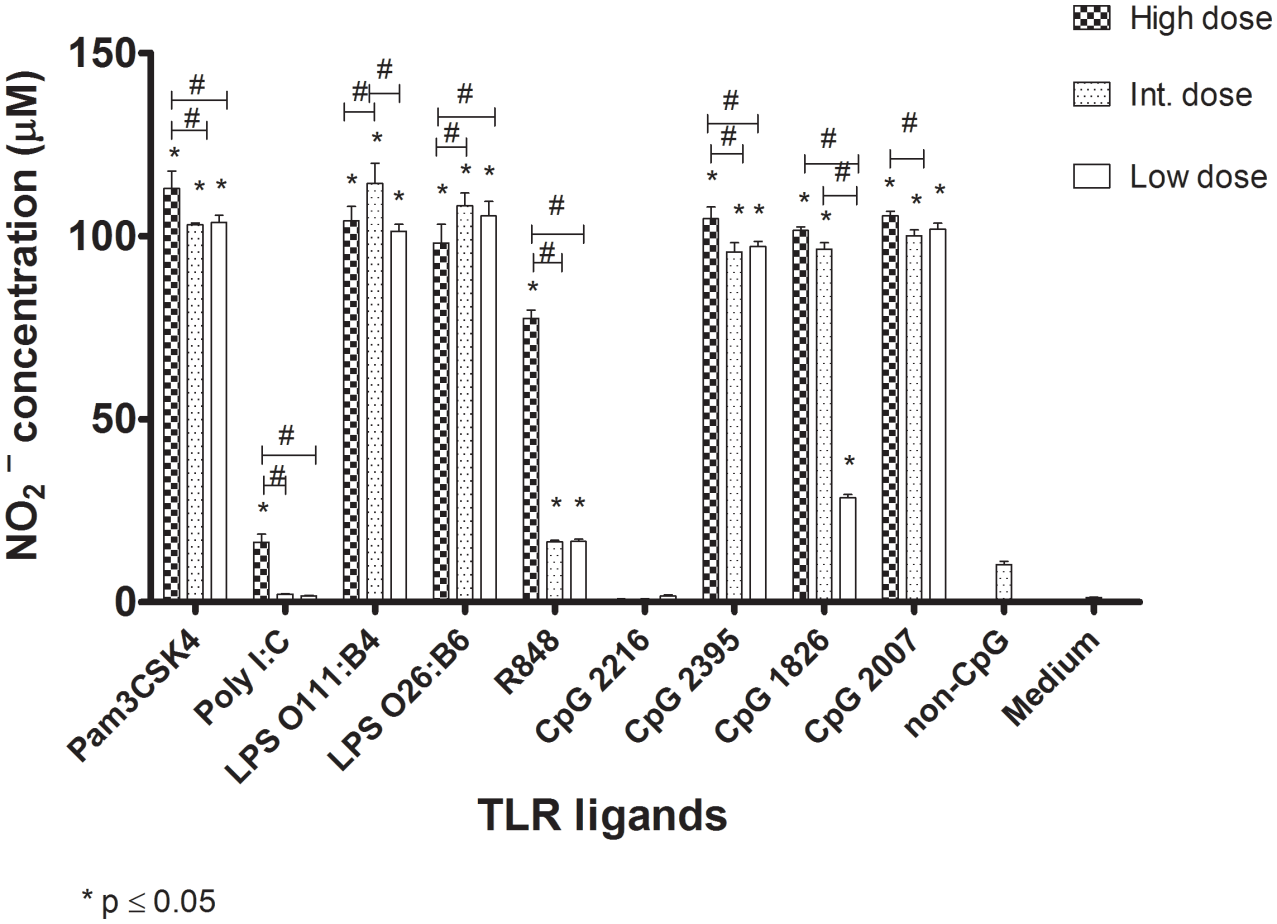
Nitrite production in chicken macrophage cells (MQ-NCSU cell line) with a variety of different types of TLR ligands. Chicken macrophages were stimulated with Pam3CSK4 (10, 1, and 0.1 µg/µl), PolyI∶C (50, 10, and 1 µg/µl), LPS (*E. coli* 0111∶B4; 10, 1, and 0.1 µg/µl), LPS (*E. coli* 026∶B6; 10, 1, and 0.1 µg/µl), R848 (10, 1, and 0.1 µg/µl), class A CpG ODN 2216 (10, 1, and 0.1 µg/µl), class B CpG ODN 2007 (10, 1, and 0.1 µg/µl), class B CpG ODN 1826 (10, 1, and 0.1 µg/µl), class C CpG ODN 2395 (10, 1, and 0.1 µg/µl), non-CpG ODN (10 µg/µl), and medium. Subsequently, nitrite in supernatants was measured after 48 hours of stimulation via the Griess assay. Nitric oxide production in each TLR ligand concentration group was compared to the cells without stimulation using a two tailed student's t test. Significant differences (*P* ≤ 0.05) between a test group and the group without stimulation (medium) are indicated by *. Significant differences (*P* ≤ 0.05) between different doses of TLR ligands are presented by #.

To examine the effects of TLR ligands on chicken macrophages, cells were treated with three different doses of ligands for TLR2, 3, 4, 7 and 21. All TLR ligands except class A CpG ODN 2216 induced a significant increase in NO production by chicken macrophages compared to the cells without TLR ligand stimulation (Fig. 2). The high dose of Pam3CSK4, polyI∶C, R848, class B CpG ODNs 2007and 1826 and class C CpG ODN 2395 induced the highest amount of NO production by chicken macrophages compared to the other doses of the corresponding TLR ligands. The intermediate dose of LPS (1 µg/µl) induced higher NO production by macrophages compared to either the high or low dose of LPS (10 µg/µl). There was no significant difference in NO production by the cells that received either LPS derived from *E. coli* 0111∶B4 or LPS from *E. coli* 026∶B6. Furthermore, there was no significant difference in NO production between the cells that received either class B CPG ODNs 2007 and 1826 or class C CpG ODN 2395, while cells treated with class A CpG ODN 2216 did not significantly produce NO compared to the cells without TLR ligand stimulation (Fig. 2). The optimal doses of TLR ligands were selected for the next experiment based on the results of this experiment. These were the ligand doses that induced the most amount of activation in macrophages, manifested by NO production.

### Pam3CSK4, LPS and CpG ODNs decrease viral replication in chicken macrophages

To determine the kinetics of response, seven different time points (1, 6, and 12 hours prior to infection, time of infection, and 1, 6, and 12 hours post-infection) for TLR ligand administration were selected and the virus titre in supernatants were initially determined using a HA assay (Table 3). PolyI∶C, R848, and class A CpG ODN 2216 did not significantly alter the HAUs in macrophage supernatant compared to cells without TLR ligand treatment. The HAUs of cells that received either Pam3CSK4 prior to (1, 6 and 12), after (1 or 6 hours) or at the time of infection were significantly lower than the untreated cells. Treatment of cells with LPS from either *E. coli* 0111∶B4 or *E. coli* 026∶B6 at 1 and 6 hours prior to infection or 1 hour post-infection had significantly lower HAU compared to untreated cells (*P* < 0.05). Stimulation of chicken macrophages with class B CpG ODNs 2007 and 1826 or class C CpG ODN 2395, 1 or 6 hours prior to infection significantly reduced HAUs in supernatants, while stimulation of chicken macrophages with either class B CpG ODN 2007 at the time of infection or class B CpG ODN 1826 12 hours prior to infection, significantly reduced HAUs in supernatants. The lowest HAU was recorded by treating the macrophages at 6 hours prior to infection with Pam3CSK4, CpG ODNs (2007, 1826 and 2395), and LPS from either *E. coli* 0111∶B4 or *E. coli* 026∶B6 and was therefore used for subsequent studies.

**Table 3:** AIV titres in supernatants of MQ-NCSU cells following treatment with TLR ligands.

TLR ligand	Hours prior to infection^a^			Simultaneously^a^	Hours post-infection^a^		
	12	6	1	0	1	6	12
Pam3CSK4	4*	3*	3.30*	3.30*	4.30*	4*	4.64
PolyI∶C	5	5	5	5	5	5	5
LPS (*E. coli* 0111∶B4)	5	3.30*	3.30*	4.64	4*	4.30*	4.64
LPS (*E. coli* 026∶B6)	5	3*	3*	4.64	4*	4.64	4.64
R848	5	4.64	4.64	4.30	5	4.64	5
CpG ODN 2216	5	5	5	5	5	5	5
CpG ODN 2007	5	3*	4*	3.30*	4.64	4.64	5
CpG ODN 1826	4.30*	3*	4*	4.64	5	5	5
CpG ODN 2395	5	3.30*	3.63*	4.64	5	5	5
Non-CpG ODN	5	4.64	3.63*	4.30*	5	5	5
Medium^C^	5	5	5	5	5	5	5

Chicken macrophages were treated with TLR ligands prior to, simultaneously, and post- infection with AIV. Supernatants were collected 14 hours post infection to measure virus titre via HA. There were four replicated for each treatment group. Titre of virus represented as Log geometric mean of haemagglutination unit (HAU) of four replicates.

a Hours relative to virus infection.

^*^Significant difference (*P* < 0.05) between treatment group and medium control group.

C Represents control group which was not stimulated with TLR ligands.

**Figure 3:**
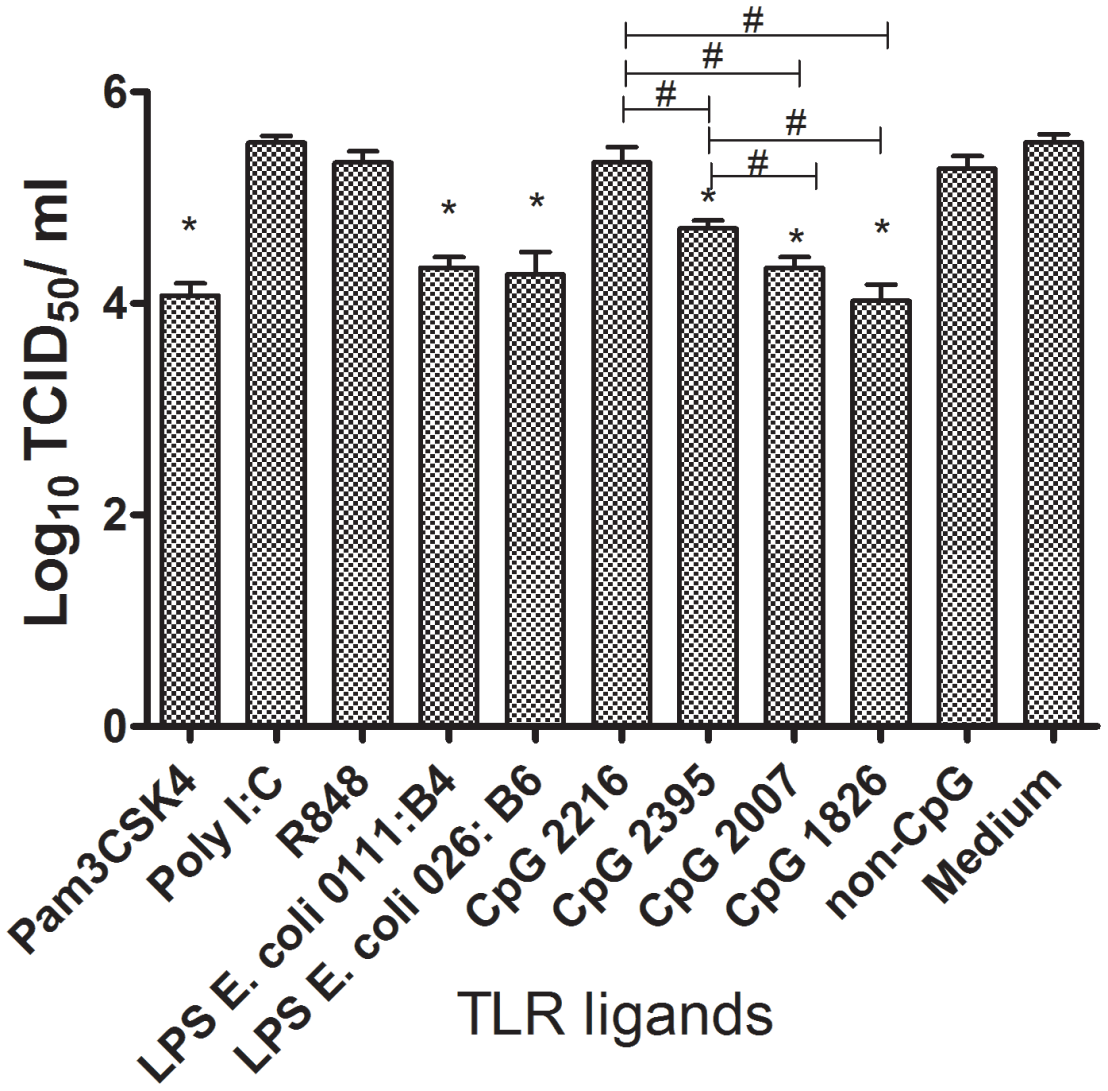
TLR ligands inhibit influenza virus replication in a chicken macrophage cell line. MQ-NCSU cells were untreated (control group) or treated with Pam3CSK4 (10 µg/µl), polyI∶C (50 µg/µl), R848(10 µg/µl), LPS from *E. coli* 0111∶B4 (1 µg/µl), LPS from *E. coli* 026∶B6 (1 µg/µl), class A CpG ODN 2216 (10 µg/µl), class C CpG ODN 2395 (10 µg/µl), class B CpG ODN 1826(10 µg/µl), class B CpG ODN 2007 (10 µg/µl) and non-CpG ODN (10 µg/µl) for 6 hours. Cells (either treated or untreated) then were infected with a MOI of 1 with H4N6 avian influenza virus for 14 hours. Virus titre was quantified via TCID_50_ assay. Each treatment group was compared to control group without TLR ligand treatment using a two tailed student's t test. Significant differences (*P* ≤ 0.05) between a test group and the group without stimulation (medium) are indicated by *. Significant differences (*P* ≤ 0.05) between different types of CpG ODN are presented by #. There were four replicates in each group. This experiment was repeated three times.

To confirm the infectivity of the virus in the supernatants, the virus titre in cells that received TLR ligand treatment 6 hours prior to infection was determined via a TCID_50_ assay. The virus titre obtained with the TCID_50_ assay was similar to that obtained with the HA assay in that the lowest virus titer was recorded by treating the macrophages with Pam3CSK4, CpG ODNs (2007, 1826, and 2395) and LPS from either *E. coli* 0111∶B4 or *E. coli* 026∶B6 (*P* ≤ 0.05; Fig. 3). Treatment of cells with polyI∶C (TLR3 ligand) and R848 (TLR7 ligand) did not significantly reduce the virus titre in the supernatants (Fig. 3). There was no significant difference between the two types of LPS in terms of their ability to reduce the virus titres. Furthermore, the supernatant from cells treated with class B CpG ODN 2007 and 1826 had significantly lower viral titres than cells treated with class C CpG ODN 2395. However, no significant difference in viral titre between cells treated with different class B CpG ODN was observed (Fig. 3).

### Induction of gene expression in macrophages by TLR ligands

**Figure 4:**
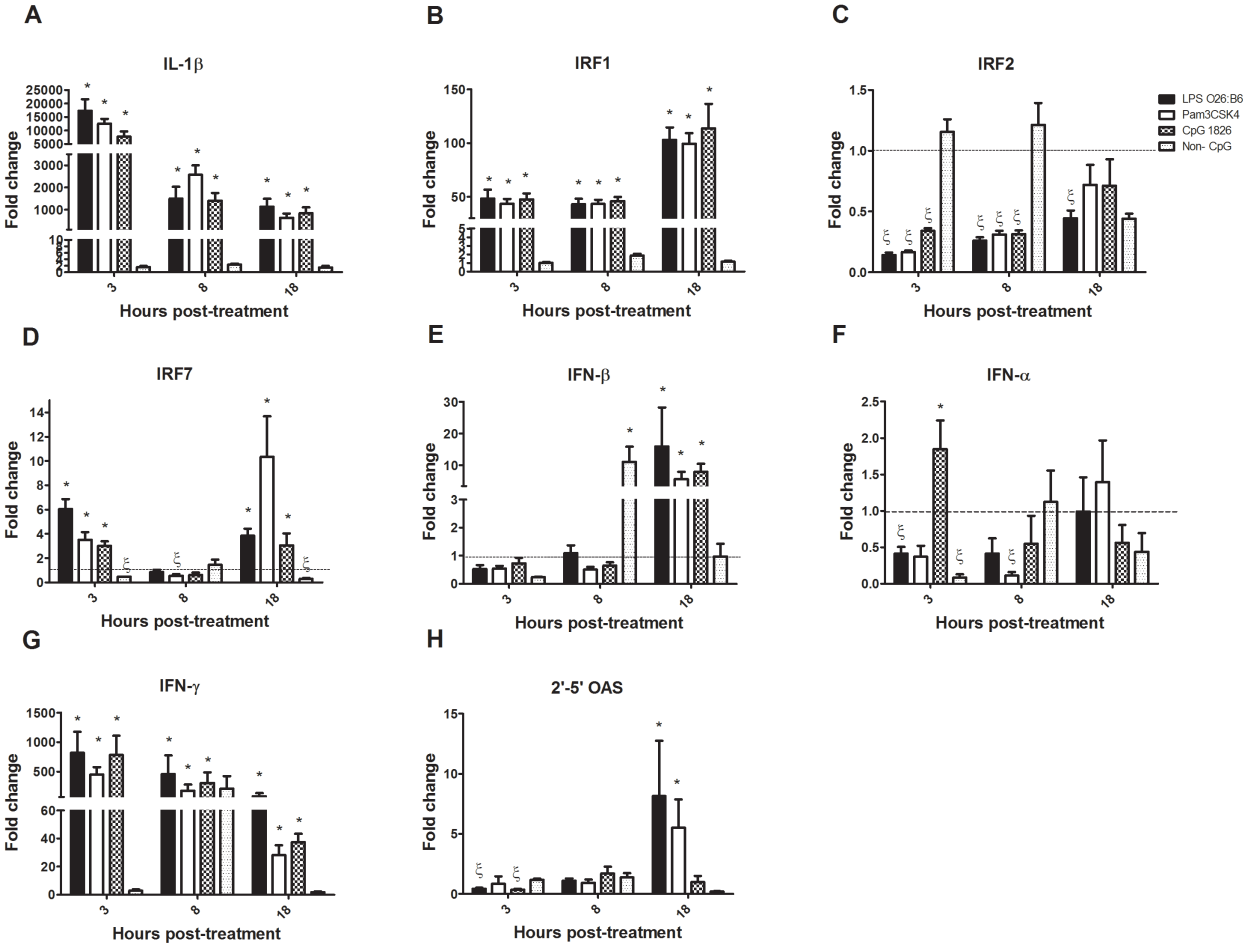
Cytokine gene expression of chicken macrophages stimulated with Pam3CSK4 (10 µg/µl), LPS from *E. coli* 026∶B6 (1 µg/µl), class B CpG ODN 1826 (10 µg/µl), and non-CpG ODN (10 µg/µl) at 3, 8 and 18 hours post-treatment. Gene expression relative to the housekeeping gene β-actin was assessed using quantitative RT-PCR. Gene expression is presented as fold changes relative to the medium control group. Error bars represent standard error of the mean. Gene expression among treatment groups was compared statistically using a two tailed student's t-distribution test. Significant up- regulation (*P* ≤ 0.05) is indicated by *, while significant down-regulation is indicated by §.

The expression of IL-1β was significantly increased in cells that were treated with Pam3CSK4 at 3, 8 and 18 hours post-treatment by 12,458, 2,570 and 638 fold, respectively (*P* ≤ 0.05). Similarly, the expression of IL-1β by macrophages incubated with 1 µg/ml of LPS was significantly increased at 3, 8 and 18 hours of incubation by 17,317, 1,490 and 1,135 fold, respectively (*P* ≤ 0.05). Moreover, class B CpG ODN 1826 caused up-regulation of IL-1β in chicken macrophages at 3, 8 and 18 hours of incubation with 7,725, 1,411 and 840 fold increases, respectively (*P* ≤ 0.05) (Fig. 4A).

The expression of interferon regulatory factor (IRF)1 in cells treated with either Pam3CSK4, LPS or class B CpG ODN 1826 was significantly increased after 3, 8 and 18 hours post-treatment (Fig. 4B). Macrophage treatment with Pam3CSK4 and class B CpG ODN 1826 caused a significant down-regulation of the IRF2 transcripts at early time points, while the expression of IRF2 in cells incubated with LPS was significantly down-regulated at 3, 8 and 18 hours of incubation (Fig. 4C). The expression of IRF7 in cells incubated with Pam3CSK4, LPS or class B CpG ODN 1826 was significantly increased at 3 and 18 hours post-treatment (Fig. 4D). In addition, the expression of IRF7 in cells incubated with Pam3CSK4 was significantly down-regulated at 8 hours of incubation by 1.88 fold (Fig. 4D).

The expression of IFN-β by chicken macrophages incubated with LPS, Pam3CSK4 and class B CpG ODN 1826 was increased at 18 hours compared to untreated cells, with 16, 5.7 and 8 fold, respectively. Moreover, the expression of IFN-β in cells incubated with non-CpG ODN was significantly up-regulated at 8 hours post-treatment with 11 fold (*P* ≤ 0.05; Fig. 4E). In addition, the expression of IFN-α by chicken macrophages incubated with class B CpG ODN 1826 was increased (1.8 fold; *P*≤ 0.05) at 3 hours of incubation compared to untreated cells (Fig. 4F). Pam3CSK4 induced a 450, 174 and 28 fold up-regulation of IFN-γ expression in macrophages at 3, 8 and 18 hours post-treatment, respectively (*P* ≤ 0.05). LPS induced an 816, 459, and 83 fold up-regulation in IFN-γ expression in macrophage cells at 3, 8 and 18 hours post-treatment, respectively (*P* ≤ 0.05). Likewise, class B CpG ODN 1826 induced a 785, 305 and 37 fold up-regulation in IFN-γ expression in macrophages at 3, 8 and 18 hours post-treatment, respectively (*P* ≤ 0.05; Fig. 4G).

The expression of 2'-5' OAS, as an ISG, by cells stimulated with LPS and class B CpG ODN 1826 was significantly down-regulated at 3 hours post-treatment (Fig. 4H). The expression of 2'-5' OAS by macrophages incubated with LPS was significantly down-regulated (2.4 fold) at 3 hours of incubation. 2′-5′OAS expression by macrophages incubated with class B CpG ODN 1826 was significantly down-regulated (2.9 fold) at 3 hours post-treatment. However, the expression of 2'-5' OAS by chicken macrophages incubated with LPS and Pam3CSK4 was increased at 18 hours of incubation compared to untreated cells, by 5.6 and 3.8 fold, respectively (*P* ≤ 0.05).

### TLR 2, 4 and 21 up-regulate macrophage activation markers

**Figure 5:**
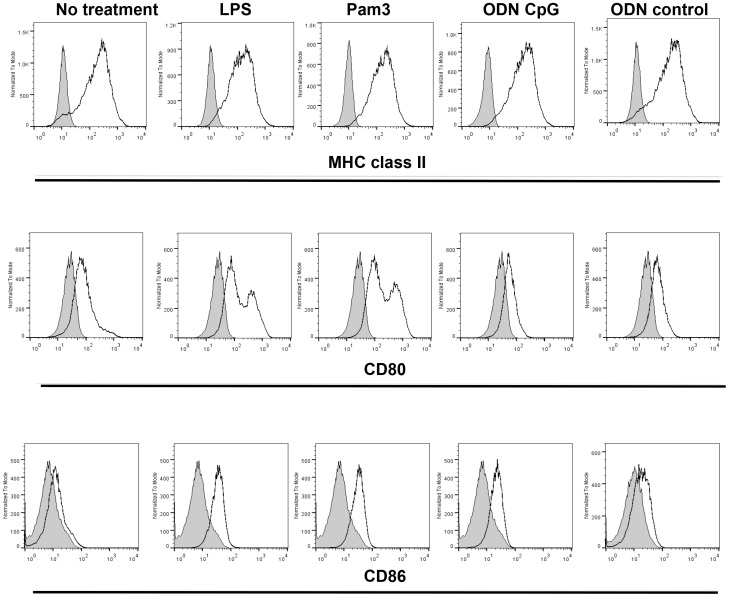
Expression levels of MHC class II, CD80 and CD86 on chicken macrophages stimulated with TLR ligands. MQ-NCSU cells were stimulated with LPS from *E. coli* 026∶B6 (1 µg/ml), Pam3CSK4 (10 µg/ml), class B CpG ODN 1826 (10 µg/ml), control ODN (10 mg/ml) or medium (no stimulation) for 24 hours. The cells were stained with FITC conjugated anti-chicken MHC class II molecules (Clone 2G11-IgG1), Alexa Fluor 647-conjugated mouse anti-chicken CD80 (clone AV82- IgG2a), Alexa Fluor 647-conjugated mouse anti-chicken CD86 (clone AV88- IgG1) or isotype controls.

We examined the effects of the TLR ligands, LPS, class B CpG ODN 1826 and Pam3CSK4, on the expression levels of MHC class II, CD80 and CD86 on chicken macrophages. The results revealed that Pam3CSK4 and LPS, but not class B CpG ODN 1826 or control ODN, up-regulated surface expression of CD80 molecules after 24 hours of stimulation (Fig. 5). The mean fluorescence intensity (MFI) of CD80 was increased by 198% in cells treated with LPS, while the cells treated with Pam3CSK4 showed 252% increase in CD80 MFI. In addition, LPS, Pam3CSK4 as well as class B CpG ODN 1826 increased (212%, 193% and 37%, respectively) surface expression of CD86. Control macrophages expressed high levels of MHC class II and TLR ligand stimulation did not alter the expression of this molecule.

## Discussion

Macrophages play an important role in the induction and regulation of immune responses and protection of the host against pathogens [45]. In particular, macrophages are involved in mounting antiviral responses [46]. Their importance is highlighted by the fact that in mammals, many viruses target macrophages and impair the function of these cells [47]–[49]. However, very little is known about the ability of avian viruses, such as AIV, to infect chicken macrophages and the consequent macrophage response to viral infection. In the present study, it was demonstrated that chicken macrophages support the replication of AIV. This study confirmed that low pathogenic H4N6 AIV can replicate within chicken macrophages and that the viral particles are infectious as demonstrated by their ability to subsequently infect MDCK cells. An increase in infectious virus was observed up to 16 hours post-infection. The results presented here are in agreement with previous studies that have demonstrated that various low pathogenic avian influenza viruses can replicate in the chicken macrophage cell line, HD11 [12], [50]. The cell culture used in the present study allowed further examination of TLR ligands potential to limit H4N6 virus replication in avian macrophages.

Pre-stimulation of macrophages with the TLR2, 4, and 21 ligands, Pam3CSk4, LPS and class B CpG ODN 1826, was marked by a reduction in H4N6 AIV replication. The timing of administration of TLR ligands however strongly influenced the ability of the cells to reduce viral replication. Although 6 hour pre-treatment was not the only time to significantly limit viral replication, it was selected for further analysis because it was the time when the greatest reduction of viral titre was observed for all three ligands. These results correlate with previous studies showing that pre-treatment with TLR7 ligands 6 hours prior to infection induced potent antiviral responses [23]. This suggests that a 6 hour pre-treatment of macrophages results in the early protection of the cells against AIV, which presumably relies on the TLR-mediated activation of antiviral responses. It has yet to be determined exactly what mechanisms play a role in the reduction of the virus replication after TLR ligand administration. However, the present findings did demonstrate that TLR ligands were able to boost the antiviral responses of macrophages, as demonstrated by an increase in expression of IRF genes, IRF1 and IRF7, type I and II IFNs, namely IFN-β and IFN-γ, and the ISG, 2′-5′ OAS. The induction and regulation of IFNs and downstream genes are crucial to establish the antiviral state in cells [51] and have been shown to be crucial against some viruses, such as influenza viruses in mammals [52], [53]. Our results suggest that similar to the case in mammals [51], chicken IRF1, 2 and 7 are involved in TLR ligand mediated signalling with the down-regulation of IRF2 and up-regulation of IRF1 and 7 initiating IFN responses that could limit H4N6 AIV replication in chicken macrophages.

Furthermore, we determined that LPS, CpG ODN and Pam3CSK4 activate chicken macrophages. Pam3CSk4 and LPS induced the expression of CD80 and CD86, while class B CpG ODN 1826 only up-regulated CD86. Although these molecules are regarded as co-stimulatory molecules that exert an important function in antigen presentation to cells of the adaptive immune system, they are also markers of macrophage activation. Therefore, we demonstrated that following stimulation with TLR2, 4 and 21 ligands, macrophages become activated, as indicated by the surface expression of CD80 and CD86 and this corresponds to the observed increase in NO production and IL-1β expression.

Previous *in vivo* studies demonstrated that CpG ODN and LPS when given prophylactically are able to reduce viral load following infection with an AIV [22]. Immunity was correlated with an increase in IFN-γ expression. In mice, swine, and humans, disease outcome has been correlated with the *in vitro* expression of IL-1β and IFN-γ [54]–[56]. Here we observed an increase in expression of IL-1β and IFN-γ in macrophages stimulated with CpG ODN, LPS, and Pam3CSk4 which coincide with the reduction in viral replication. IFNs have a critical role in limiting viral infection, for example in the case of human influenza virus [52], [57]. Therefore TLR ligands may interfere with viral replication through the induction of IFNs and downstream ISGs. The direct antiviral activity of IFN-γ against AIV in chickens has not been characterized, but IFN-γ induces nitric oxide production, macrophage cell surface markers and up-regulation of some ISGs such as 2'-5' OAS. Therefore, it might be that IFN-γ through the initiation of RNase L pathway, indirectly, interferes with the replication of AIV in macrophages [22], [58]. Furthermore, although the host response to AIV is complex, it is known that macrophages are key participants in the ability of the innate immune system to respond and subsequently activate the adaptive immune system after AIV infection. Therefore, it is possible that TLR ligand administration *in vivo* stimulates macrophages and the activated macrophages can act as antigen presenting cells and activate the adaptive immune response to influenza virus infection.

In contrast, the TLR3 ligand, poly(I∶C) reduced viral shedding *in vivo* [22] but did not stimulate chicken macrophages *in vitro* or reduce viral titres in these cells. These results suggest that stimulation of macrophages is not the only way in which TLR treatment limits viral replication *in vivo*. The lack of response observed in macrophages corresponds to previous reports that demonstrated that poly(I∶C) is a weak NO inducer in chicken monocytes [59]. Conversely, poly(I∶C) has been shown to stimulate HD11 cells, to produce NO [4], [59], [60]. Although both MQ-NCSU and HD11 are macrophage cell lines, it has been shown that their TLR3 expression, functions, responsiveness, and susceptibility to infection are different [61], [62]. The limited responses observed in this experiment may be related to the limited and variable expression of TLR3 in chicken macrophages [20], [63].

Varying effects were observed when different classes of CpG ODN were applied in this study, as shown by the production of NO by macrophages and the reduction of viral replication. Previous studies also demonstrated chicken macrophages respond differently to different types of CpG ODN in a sequence-specific manner [36]. Different levels of TLR expression and the activation of downstream signalling cascades can cause divergent and sometimes contrasting responses in cells [34], [64]. Additionally, our results demonstrate that different types of CpG ODN may exert different levels of antiviral effects. For example, class A CpG ODN 2216 treatment did not reduce viral replication, while both class B and C CpG ODN reduced viral replication in chicken macrophages. However, differences in activity of CpG ODN may also exist within the same class of CpG ODN. For instance, it has been demonstrated that class B CpG ODN 2006 is more potent at inducing pro-inflammatory cytokines, nitric oxide production and bacterial intracellular killing compared to class B CpG ODN 1826 [65].

Overall, our results demonstrated that the TLR2, 3 and 21 ligands, Pam3CSK4, LPS and CpG ODN, activated macrophages and initiated pro-inflammatory and antiviral responses in these cells. In addition, our results indicate that TLR ligands have varying abilities with respect to inducing antiviral responses against avian influenza virus in chicken macrophages. The appropriate time point for treating macrophages with TLR ligands and the dose of TLR ligand could affect the outcome of macrophage activation. Transcriptional analysis revealed that TLR2, 4 and 21 ligands were able to induce the expression of IL-1β, IFN-γ, IRF7, and IFN-β in macrophages, which might play a role in control of AIV replication in these cells. Future studies should be aimed at characterizing the antiviral properties of ISG proteins in avian cells infected with influenza viruses.
